# Characterization of the Protective Cellular Immune Response in Pigs Immunized Intradermally with the Live Attenuated African Swine Fever Virus (ASFV) Lv17/WB/Rie1

**DOI:** 10.3390/vaccines12040443

**Published:** 2024-04-20

**Authors:** Miriam Pedrera, Alejandro Soler, Alicia Simón, Nadia Casado, Covadonga Pérez, María A. García-Casado, Paloma Fernández-Pacheco, Pedro J. Sánchez-Cordón, Marisa Arias, Carmina Gallardo

**Affiliations:** 1 Centro de Investigación en Sanidad Animal (CISA), Instituto Nacional de Investigación y Tecnología Agraria y Alimentaria (INIA), Consejo Superior de Investigaciones Científicas (CSIC), Valdeolmos, 28130 Madrid, Spain; 2 European Union Reference Laboratory for African Swine Fever (EURL), Centro de Investigación en Sanidad Animal (CISA), Instituto Nacional de Investigación y Tecnología Agraria y Alimentaria (INIA), Consejo Superior de Investigaciones Científicas (CSIC), Valdeolmos, 28130 Madrid, Spain

**Keywords:** African swine fever, live-attenuated virus, vaccine, protective cellular response, T-cell, cytokines, domestic pigs

## Abstract

Candidate vaccines against African swine fever virus (ASFV) based on naturally attenuated or genetically modified viruses have the potential to generate protective immune responses, although there is no consensus on what defines a protective immune response against ASFV. Studies, especially in sensitive host species and focused on unravelling protective mechanisms, will contribute to the development of safer and more effective vaccines. The present study provides a detailed analysis of phenotypic and functional data on cellular responses induced by intradermal immunization and subsequent boosting of domestic pigs with the naturally attenuated field strain Lv17/WB/Rie1, as well as the mechanisms underlying protection against intramuscular challenge with the virulent genotype II Armenia/07 strain. The transient increase in IL-8 and IL-10 in serum observed after immunization might be correlated with survival. Protection was also associated with a robust ASFV-specific polyfunctional memory T-cell response, where CD4CD8 and CD8 T cells were identified as the main cellular sources of virus-specific IFNγ and TNFα. In parallel with the cytokine response, these T-cell subsets also showed specific cytotoxic activity as evidenced by the increased expression of the CD107a degranulation marker. Along with virus-specific multifunctional CD4CD8 and CD8 T-cell responses, the increased levels of antigen experienced in cytotoxic CD4 T cells observed after the challenge in immunized pigs might also contribute to controlling virulent infection by killing mechanisms targeting infected antigen-presenting cells. Future studies should elucidate whether the memory T-cell responses evidenced in the present study persist and provide long-term protection against further ASFV infections.

## 1. Introduction

African swine fever (ASF) is currently the main problem the pig industry is facing worldwide [1]. ASF is a devastating hemorrhagic infectious disease caused by African swine fever virus (ASFV), a large and complex enveloped double-stranded DNA virus of the genus Asfirvirus (family Asfarviridae). The disease was endemic in most sub-Saharan African countries and on the island of Sardinia (Italy) until 2007, when highly virulent isolates belonging to genotype II appeared in Eastern Europe. Since then, ASF has become endemic in many European and Asian countries spreading without control into Eastern Europe, China (since 2018) and most Southeast Asian countries, causing a huge economic impact. Outbreaks of uncertain origin have also occurred since 2021 in some Caribbean countries (Dominican Republic and Haiti), posing a threat to the nearby North American pork industry [2]. The disease, which affects domestic and wild suids of all breeds and ages, presents a variable lethality depending on the virulence of the isolate and the immune status of the infected animals [3,4]. Thus, highly virulent isolates often have a lethality close to 100% in naïve animals, which usually die within two weeks of infection [5].

Vaccines are essential to control viral diseases. The lack of vaccines against ASFV can largely be attributed to gaps in knowledge of the strategies used by the virus to evade host innate and adaptive immunity and the functions of virus proteins responsible for inducing protective immune responses [6]. Evidence suggests the importance of both arms of adaptive immunity for protection. However, the immunological correlates of protection are not yet understood. On the one hand, antibodies have been suggested to be an essential component of protective immunity against virulent ASFV, although alone they are not sufficient to induce protection [7]. Conversely, evidence suggests that cellular immune response, even in the absence of specific antibodies against ASFV, may be necessary for effective protection, highlighting the role of CD8+ T lymphocytes [8,9,10,11,12,13,14]. The clearest evidence of the relevance of CD8+ T lymphocytes in protection was demonstrated after in vivo depletion of CD8α+ T cell in ASFV-immune pigs, which led to the loss of the protective capacity conferred by immunization with an attenuated ASFV strain [8]. More recent studies have also demonstrated the important role of T-cell responses in protection, suggesting that both cytotoxic CD8+ T cells and memory CD4+CD8+ T cells may play a key role in the protective immunity conferred by vaccination [11,12,14,15,16].

Vaccines against ASFV based on a naturally attenuated live virus (LAV) induce robust immune protection, stimulating both innate and adaptive (cellular and humoral) immunity. LAV immunizations, although characterized by an absence or mild presence of both clinical signs and viremia levels, have traditionally raised safety concerns, making their commercialization unlikely [6,17]. Selective deletion of genes involved in virus attenuation and/or induction of protection has also been used as a strategy to produce safe and effective live attenuated vaccines and to differentiate between vaccinated and infected animals (DIVA vaccines). In both cases, viruses attenuated naturally or by gene deletion usually render recovered animals protected from subsequent infections with related viruses, although this does not usually guarantee protection against divergent viruses [17,18,19,20]. Previous studies have demonstrated that immunization of domestic pigs (DPs) and wild boar with a naturally attenuated non-hemadsorbing (non-HAD) genotype II ASFV isolate, obtained from a wild boar hunted in Latvia in 2017 (Lv17/WB/Rie1 strain), conferred high levels of protection against challenges with a virulent ASFV genotype II isolate (Armenia/07) [21,22,23]. It is known that immunizations with this strain induce some mild clinical signs and transient viremia. To improve its safety, the deletion of virulence-associated genes has recently been carried out. Although some deletion mutants generated showed a slight reduction in pathogenicity and lethality during the in vivo evaluations in DP, they did not show a significant reduction in side effects with respect to the parental virus [24,25]. These results highlight the potential use of the Lv17/WB/Rie1 strain not just as a vaccine prototype, but also as an excellent platform to elucidate protective mechanisms in immunized animals with special attention given to the role of the cellular immune response. With the objective of characterizing the immunological mechanisms of protection against ASFV, and with special attention on the role played by different subsets of T lymphocytes, we have systematically analyzed these mechanisms in DP immunized with the genotype II Lv17/WB/Rie1 strain and protected against virulent challenge with Armenia/07 (Arm07). In addition, several immunomodulatory cytokines were studied to complete the understanding of the protective mechanisms elicited by this vaccine prototype.

## 2. Materials and Methods

### 2.1. Viruses and Cells

The naturally attenuated, non-hemadsorbing (non-HAD) genotype II ASFV strain Lv17/WB/Rie1 was used for the immunization of domestic pigs. The hemadsorbing (HAD) ASFV strain Armenia/07 (Arm07), a virulent genotype II strain [26], was used to challenge the animals. Lv17/WB/Rie1, isolated for the first time from the serum of a wild boar hunted in Latvia in 2017, was previously described and tested in experimental trials in domestic pigs [23] and wild boar [21]. For the in vivo immunization/challenge studies, Lv17/WB/Rie1 was propagated in porcine alveolar macrophage (PAM) cultures, while Arm07 was propagated and titrated in porcine blood leukocytes (PBLs) as described previously [27]. Titers of virus were defined as the amount of virus causing HAD (for the HAD strain) or infection, assessed by immunoperoxidase (IP) staining (for non-HAD strain), in 50% of infected cell cultures (HAD_50_/mL or TCID_50_/mL, respectively). For the ex vivo stimulation assays, Lv17/WB/Rie1 and Arm07 were propagated in PAM, stocks were titrated in 48-well plates using the African green monkey fibroblast-like cell line (Cos-7 cells cells), and viral titers (TCID_50_/mL) were estimated by IP staining. Mock virus supernatant was prepared from uninfected PAM and stock was titrated in the same way as virus stocks.

Cos-7 cell culture was originally obtained from the American Type Culture Collection (ATCC CRL-1651) and it was grown in Dulbecco’s modified Eagle Medium (Corning, Manassas, VA, USA) supplemented with 2 mM L-glutamine, 100 U of gentamicin per mL (Sigma-Aldrich, Saint Louis, MO, USA), 1% Na Pyruvate (Thermo Scientific, Waltham, MA, USA) and 1% non-essential amino acids (Thermo Scientific, Waltham, MA, USA). Cells were cultured at 37 °C in medium supplemented with heat inactivated (HI) fetal bovine serum (FBS) (Sigma-Aldrich, Saint Louis, MO, USA).

The MS-stable monkey kidney cell line (ECACC, 91070510) was used for the preparation of the ASFV MS-adapted E70 isolate (E70 MS 48); coated 96-well plates. These plates were then used as the antigen in the indirect IP test (IPT) [28].

### 2.2. Animals and Experimental Design

The in vivo experiments were conducted under biosafety level 3 (BSL3) conditions at the animal facilities of Centro de Investigación en Sanidad Animal (CISA-INIA-CSIC), in accordance with EC Directive 2010/63/UE and approved by the Spanish Ethical and Animal Welfare Committee (Ref nº PROEX/101/8.21). Nine ASFV-free and ASFV antibody-free 8-week-old European hybrid females were randomly allocated to two groups. Animals in one group (n = 5; pig 6, 7, 8, 9 and 10) were immunized on day 0 (prime) by intradermal (ID) inoculation using the IDAL 3G+ needleless vaccinator (IDAL^®^, MSD Animal Health, Rahway, NJ, USA) with 1 mL containing 10^2^ TCID_50_ of the attenuated ASFV Lv17/WB/Rie1 strain. These animals received identical immunization on day 21 (boost). Animals in the other group (n = 4) remained as non-immunized controls. Thirty-five days after the first immunization, the pigs were challenged by the intramuscular (IM) route with 1 mL containing 10 HAD_50_ of the virulent Arm07 strain. In the control group, non-immunized animals were challenged in parallel using the same route and dose.

### 2.3. Sampling, Clinical and Post-Mortem Analysis

For this study, blood samples were taken from all pigs on a weekly basis. Serum was collected in BD SST vacutainer tubes (Fisher Scientific, Hampton, NH, USA). Anticoagulated blood was collected in heparin tubes for peripheral blood mononuclear cell (PBMC) isolation and in EDTA for virological studies (BD vacutainer tubes, Fisher Scientific). Clinical signs were recorded daily post-immunization (dpi) and post-challenge (dpc) and expressed with a quantitative clinical score by summing the values of eight clinical signs as previously described [29]. Parameters such as hyperthermia, loss of appetite, recumbence, skin hemorrhage or cyanosis, joint swelling, respiratory distress, ocular discharge, and digestive disturbances were assigned points on a severity scale of 0 to 3, with one being mild, two being moderate, and three the most serious. The sum of the points was recorded as the clinical score (CS), which was also used to define humane endpoints. Pre-determined humane endpoints included pigs displaying severe clinical signs such as hyperthermia, anorexia, recumbence, respiratory distress and digestive disturbances for more than two consecutive days, or a total CS in a pig of >18. Pigs in the immunized group were euthanized on day 65 after prime immunization (30 days after challenge) for tissue collection. Non-immunized pigs were culled from 7 dpc after reaching a specified humane endpoint. Macroscopic lesions were evaluated during necropsies, using scores based on previous standardized protocols [30]. Twenty-one different types of tissues and organs were obtained from each necropsied animal and frozen at −80 °C for ASFV genome detection. 

### 2.4. Sample Processing

Serum tubes were stored at −80 °C for further analysis. PBMCs were isolated from heparinized blood by density gradient centrifugation as described previously [31]. PBMCs were resuspended in complete RPMI 1640 medium (cRPMI) supplemented with 10% HI FBS and antibiotics and were used immediately at the cell density required for the different immunological assays or cryopreserved for subsequent analysis. Organ and tissue samples were homogenized using stablished protocols [32]. Briefly, 10% (*w*/*v*) clarified homogenized tissue suspensions were prepared in PBS. Supernatants were filtered with MINISART filters 0.45 µm and then treated with 0.1% of gentamicin sulphate 50 mg/mL (BioWhittaker, Walkersville, MD, USA) for 1 h at 4 ± 3 °C prior to use for virus detection. 

### 2.5. Assessment of the Presence of Virus in Blood and Tissues

DNA was isolated from EDTA-blood and homogenized tissue samples using the High Pure PCR Template Preparation kit (Roche Diagnostics GmbH, Roche Applied Science, Mannheim, Germany) and the Universal Probe Library (UPL) real-time PCR (PCR) [32] was carried out for each sample. ASFV-positive samples were those with a Ct < 40. Virus isolation (VI) was performed on PCR-positive blood and tissue samples using PBL in 96-well plates as described in the *WOAH Manual* [32]. The plates were examined over a period of seven days to evidence the presence of HAD or cytopathic effect (CPE). In the absence of HAD, the samples were blind passaged three times and subjected to the UPL PCR [32] to verify the replication of the non-HAD Lv17/WB/Rie1 ASFV strain. Titers were estimated by end-point dilution as described previously in Section 2.1. 

### 2.6. Assessment of ASFV-Specific Antibodies and Cytokines in Serum

Sera were assayed for ASFV-specific antibodies (Abs) using a commercial ELISA kit (Ingenasa-Ingezim PPA Compac K3; Gold Standard Diagnostic, Madrid, Spain). Antibody titers were determined by end-point dilution using the IPT [28,32] and expressed as the reciprocal of the highest dilution showing a positive result. Commercial ELISA kits were used to analyze serum concentrations of different cytokines with important immunoregulatory functions according to the manufacturer’s instructions: TNFα, IFNγ, IL-8, IL-10, IL-4, IL-12 and IL-6 (R&D Systems, Abingdon, UK) and IFNα (Millipore-Sigma, Saint Louis, MO, USA). For cytokine analysis, serum samples collected at the same time points during the course of the study from immunized pigs, and non-immunized control pigs were used to compare the different cytokine profiles. Only serum samples collected before the challenge from two of the four pigs included in the control group were analyzed in the cytokine ELISA assays, while serum samples collected after the challenge from all animals in this group were included in the assay.

### 2.7. Assessment of ASFV-Specific T-Cell Cytokine Responses in Blood

#### 2.7.1. IFNγ ELISpot Assay

A broad assessment of virus specific T-cell responses was carried out by longitudinal measurement of IFN-γ secretion using a porcine ELISpot assay [31,33]. IFN-γ responses to antigenic stimulation were assessed after in vitro stimulation of PBMC at 0, 7, 21, 28, 35, 42, 49 and 56 dpi, either with Lv17/WB/Rie1 or Arm07. A total of 2.5 × 10^5^ cells were added per well to the pre-coated ELISpot plates (Millipore, Watford, UK) and tested in triplicated wells at a multiplicity of infection (MOI) of 0.1 for each virus. Mock virus-infected PAM cell supernatant and concanavalin A (ConA) (Sigma-Aldrich) were used as negative and positive controls, respectively. Spots were visualized and counted using an automated ELISpot reader (AID AutoImmun Diagnostika, Straβberg, Germany) and results were expressed as the mock-corrected number of IFN-γ secreting cells or spot-forming units (SFUs) per million PBMC.

#### 2.7.2. Flow Cytometric Analysis

In order to characterize the phenotype and functions of virus-specific T cells a flow cytometry analysis was performed [31,33,34]. Cryopreserved PBMCs from selected days (0, 7, 21, 28, 35, 42, 49 and 56 dpi) were recovered from liquid nitrogen storage and resuscitated in pre-warmed cRPMI. A total of 5 × 10^5^ cells were added per well into 96-well round-bottom plates (Nunc, Thermo Scientific). Cells were stimulated in triplicate with 0.1 MOI of virus using either Lv17/WB/Rie1 or Arm07. The medium only or the mock-virus supernatant were used as negative controls. In the positive control wells, a cell activation cocktail containing phorbol 12-myristate-13-acetate and ionomycin (PMA/Ionomycin) (BioLegend, San Diego, CA, USA) was used. Brefeldin A (GolgiPlug, BD Biosciences, Franklin Lakes, NJ, USA) was added after 14–16 h at 37 °C and cells were incubated for another 6 h. In defined experiments, CD107a-Alexa Fluor 647 monoclonal Ab (mAb) (Bio-Rad, Kidlington, UK), in conjunction with Brefeldin A (GolgiPlug) and Monensin (GolgiStop) (BD Biosciences), were used to study cytotoxic degranulation. Prior to intracellular cytokine staining (ICS), cells were surface stained with Zombie NIR fixable viability dye (Biolegend) and with the mAbs CD3ε-FITC, CD4α-PerCP-Cy5.5 and CD8α-PE (BD Bioscience). At selected time points during the experiment and in specific laboratory assays, mouse anti-pig swine leukocyte antigen (SLA) class II DR mAb (Bio-Rad) was added to study cell activation. Following fixation and permeabilization, cells were labelled with IFN-γ- Alexa Fluor 647 (Bio-Rad) and TNFα-Brilliant Violet 421 (Biolegend) and analyzed using a BD FACSCelesta Cell Analyzer (BD Biosciences). This analysis was carried out by the selection of lymphocytes, followed by the selection of singlet and live events. Then CD3+CD4+CD8^int/low^ (CD4+CD8+ T cells), CD3+CD4^neg^CD8^high^ (CD8+ T cells) and CD3+CD4+CD8^neg^ (CD4+ T cells) cell subpopulations were gated and their frequencies relative to the live cells analyzed. Moreover, frequencies of cells positive to IFNγ, CD107a or SLA class II (SLA-II), as well as co-expression of TNFα, were analyzed for all those subpopulations. Mean frequencies of the different cell populations in the mock-stimulated wells were subtracted from each experimental value of each animal. Likewise, the mean % of IFNγ+ or IFNγ+TNFα+ secreting cells, and the mean % of CD107a+ or CD107a+TNFα+ cells, as well as the mean % of SLA-II+ or SLA-II+ TNFα+ cells in the mock-stimulated wells were subtracted from each experimental value of each animal in order to analyze the virus-specific cellular responses. The numbers of singlet live lymphocytes acquired for analysis ranged from 30,000 to 50,000 events.

### 2.8. Statistical Data Analysis

For graphical and statistical analysis of data, the software GraphPad Prism v8.0.1 (GraphPad Software, San Diego, CA, USA) was used. Flow cytometry data were analyzed using FlowJo v10 (BD). In order to compare virus-specific cellular responses from 0 dpi onwards in the immunized group and between groups and/or virus stimulus at different time points, a two-way analysis of variance (ANOVA) or a mixed-effects model was used. Antibody titer data were log transformed before analysis. *p*-values < 0.05 were considered statistically significant.

## 3. Results

### 3.1. Evaluation of Immunization Efficacy

#### 3.1.1. Clinical Signs and Viremia 

**Figure 1:**
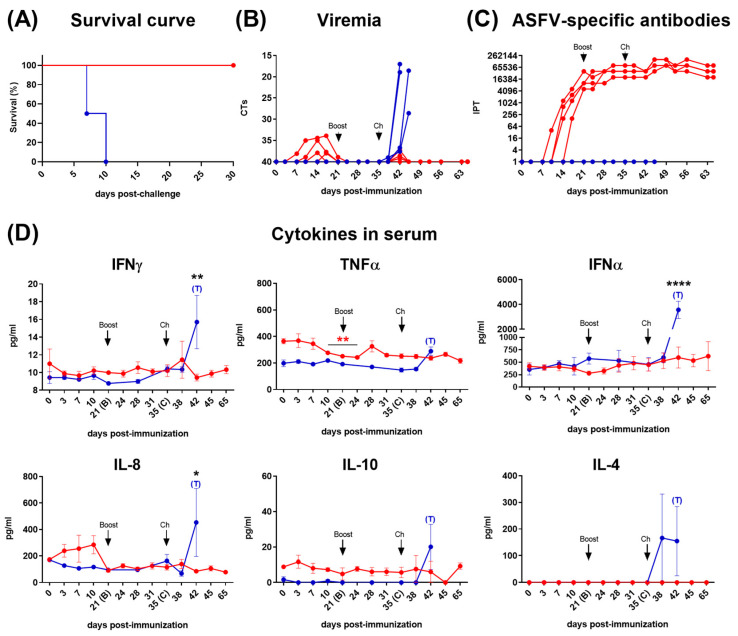
Kaplan-Meier survival curve comparing both groups after challenge with the virulent Armenia07 strain (**A**). Viremia (**B**), ASFV-specific antibody response (**C**), and cytokines in serum samples (**D**) following immunization of pigs on day 0 (prime) and 21 (boost) dpi with Lv17/WB/Rie1 and challenge at 35 dpi with Armenia07. Immunized pigs (red line) and non-immunized pigs (blue line). Values at each time point for each individual pig are shown (**B**,**C**). Mean data ± SEM are shown for each group (**D**). Significance is indicated by the following: * (*p* < 0.05), ** (*p* < 0.01), *** (*p* < 0.001) and ****(*p* < 0.0001). Black asterisks indicate significance between groups (“matched time point”). Significance within group between day 0 and the different days post-immunization is indicated by red asterisks (immunized group) or blue asterisks (non-immunized control group).

All the pigs in the immunized group were successfully immunized with Lv17/WB/Rie1. Except for one pig (pig 7), the animals showed no or mild clinical signs, and remained healthy across the entire observation period. After the prime immunization, pig 7 developed moderate clinical signs, showing fluctuating hyperthermia between 10 and 14 dpi (with a peak up to 41.6 °C), which coincided with moderate levels of viremia (Ct values from 26.3 to 25.6). Consequently, pig 7 (with a mean CS of 12) was euthanized at 14 dpi and it was excluded from further immunological evaluations. The remaining immunized animals exhibited mild clinical signs (mean CS 2.4 ± 0.9) with low and sporadic viremia (Ct values above 34) between 7 and 21 dpi (Figure 1B). Excluding pig 7, virus was solely isolated from blood samples obtained from pig 10 at 10 and 14 dpi, with an average viral titer of 9.8 × 10^2^ TCID_50_. After the challenge with the virulent Arm07 strain, all animals in the immunized group survived throughout the entire experiment, without apparent clinical signs and with barely detectable virus levels in the blood (Ct values above 37.3) at 42 dpi (7 dpc). No infectious virus was recovered by virus isolation from the blood of any of the immunized pigs after the challenge. On the contrary, non-immunized control pigs became severely ill and were humanely culled from 7 days after the challenge with Arm07, showing clinical signs and viremia levels characteristic of acute ASF. A Kaplan–Meier survival plot for both groups after the challenge is shown (Figure 1A).

#### 3.1.2. Pathological Finding and Presence of Virus Genome in Tissues

The detection of the ASFV genome by PCR in tissue samples taken during necropsies of unvaccinated (7 dpc) and immunized pigs (30 dpc) is shown in Table 1. Non-immunized control pigs displayed characteristic ASF lesions after the challenge. Tissue samples were collected from two randomly chosen control pigs, identified as #11 and #15, to evaluate viral load. The ASFV genome was detected in all tissues, with an average Ct value of 20.56 ± 0.8 (Table 1). Virus isolation was successful from all samples, with mean titers ranging from 4.03 × 10^6^ HAD_50_/mL in intra-articular tissues of joints to 4.55 × 10^8^ HAD_50_/mL in target tissues such as liver, lung, spleen, tonsil, and lymph nodes. In contrast to this, no such lesions were observed in immunized animals during necropsies carried out at the end of the study (65 dpi/30 dpc). The ASFV genome was found in 6/21 tissues (28.6%) in pig 6, 1/21 (4.8%) in pig 8, 5/21 (23.8%) in pig 9 and 14/21 (66.6%) in pig 10. The mean average Ct value was 36.34 ± 2.45. Lv17/WB/Rie1 was isolated from some tissues (lymph nodes and articular cartilage) obtained from two immunized pigs (pig 6 and pig 10) with an average titer of 1.16 × 10^2^ TCDI_50_/mL, whereas Arm07 was isolated from the palatine tonsil in one of the immunized pigs (pig 10) with a titer of 3.58 × 10^3^ HAD_50_/mL (Table 1).

### 3.2. Evaluation of ASFV-Specific Antibodies and Cytokine Profiles in Serum

All pigs seroconverted from 12 ± 1.6 dpi, showing a robust response from 21 dpi, which was maintained until the end of the study (Figure 1C). Serum levels of key immunoregulatory cytokines were monitored throughout the study (Figure 1D). Immunization with Lv17/WB/Rie1 did not result in a significant increase in serum IFNγ levels. However, although barely perceptible, we observed small increases in this cytokine in some of the immunized animals, displaying a first peak 7 days after booster immunization (28 dpi) and a second and more marked increase 3 days after the challenge (38 dpi). TNFα levels exhibited a gradual decrease following primary immunization, achieving significant differences regarding pre-immunization values between 10 and 24 dpi (*p* < 0.05). At 28 dpi, seven days after the booster immunization, a non-significant but punctual increase in TNFα was detected. Other important pro-inflammatory cytokines, such as IFNα, did not show significant changes at any time in the immunized group. However, between 10 and 24 dpi, in parallel with the TNFα, a slight decrease in IFNα was also observed. Conversely, a remarkable, albeit not statistically significant, increase in serum levels of IL-8 (also known as CXCL-8) was observed between 3 and 10 dpi. IL-10 levels remained generally low throughout the study, with a slight, non-significant peak at 3 dpi, while IL-4 was undetectable at any time point. Finally, other important cytokines, such as IL-12 or IL-6 did not exhibit appreciable changes in the immunized animals. By contrast, following the challenge, serum levels of all studied cytokines increased in the non-immunized control pigs, displaying some significant differences with the immunized animals, particularly notable for IFNγ (*p* < 0.01) and IFNα (*p* < 0.0001), and slightly lower for IL-8 (*p* < 0.05).

### 3.3. Evaluation of ASFV-Induced Protective Cellular Responses after Immunization with the Lv17/WB/Rie1 Strain and Homologous Virulent Challenge with Armenia/07

#### 3.3.1. Analysis of Virus-Specific IFNγ Responses by IFNγ T Cell ELISpot

**Figure 2:**
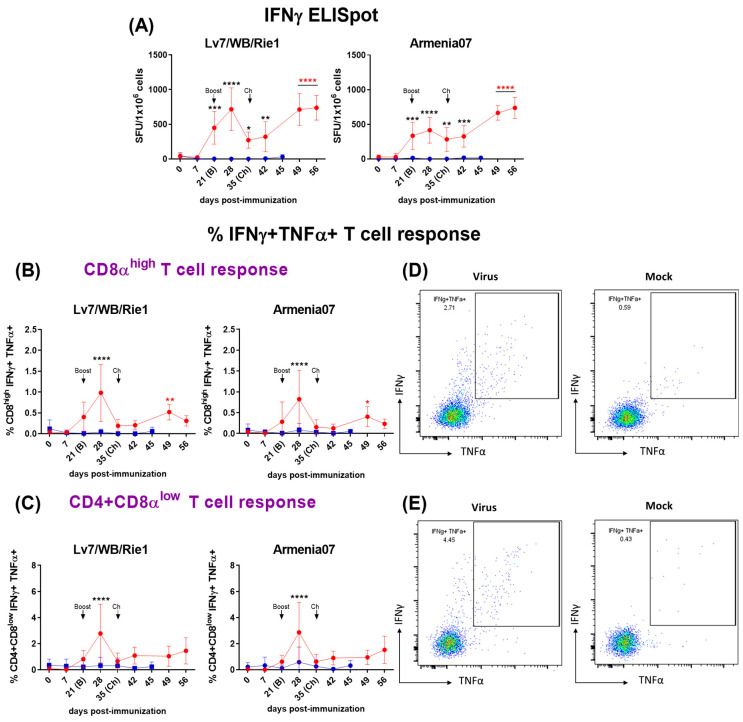
(**A**) Evaluation by IFNγ ELISpot of virus-specific IFNγ T-cell responses following immunization with Lv7/WB/Rie1 on 0 (prime) and 21 (boost) dpi and challenge with Armenia07 at 35 dpi. Responses of PBMC to stimulation with Lv7/WB/Rie1 and Arm07 are presented as the mock-corrected number of IFNγ spot-forming units (SFUs) per million cells. (**B**–**E**) Phenotyping of virus-specific IFNγ responder cells by intracellular cytokine staining (ICS) using flow cytometry following immunization with Lv7/WB/Rie1 on 0 (prime) and 21 (boost) dpi and challenge with Armenia07 at 35 dpi. T-cell populations were defined as CD3+CD4^neg^CD8^high^ (cytotoxic CD8+) (**B**) and CD3+CD4+CD8^low^ (memory CD4+CD8+) (**C**). Intracytoplasmic co-expression of IFNγ and TNFα was assessed in each population. Longitudinal responses of resuscitated PBMCs to stimulation with Lv7/WB/Rie1 and Armenia07 are presented as the mock-corrected %IFNγ+TNFα+CD8α^high^ (**B**) and %IFNγ+TNFα+CD4+CD8α^low^ T cells (**C**). Immunized group (red line) and non-immunized control group (blue line). Mean data ± SD from four pigs/time points are shown for each group. (**D**) Representative dot plots showing gates defining double expression of IFNγ+TNFα+ in singlet, live cytotoxic CD8+ T lymphocytes after stimulation with virus or mock. (**E**) Representative dot plots showing gates defining double expression of IFNγ+TNFα+ in singlet, live memory CD4+CD8+ T lymphocytes after stimulation with virus or mock. Representative dot plots from one pig of the immunized group at 28 dpi are displayed. Final gates define percentages of double expression %IFNγ+TNFα+. Significance is indicated by the following: * (*p* < 0.05), ** (*p* < 0.01), *** (*p* < 0.001) and **** (*p* < 0.0001). Black asterisks indicate both significance between groups (“matched time point”) and significance with respect to day 0 within the group of immunized pigs. Red asterisks indicate only significance with respect to day 0 within the group of immunized pigs.

Virus-specific T-cell response was quantified throughout the experiment using an IFNγ ELISpot assay after ex vivo stimulation of PBMC using either Lv17/WB/Rie1 or Arm07 (Figure 2A). The immunization of pigs with Lv17/WB/Rie1 induced a strong virus-specific IFNγ T cell response, which was observed following the prime immunization, with significant increases (*p* < 0.001) in the responding cells against both viruses (Lv17/WB/Rie1 and Arm07) at 21 dpi. Both responses were even greater after the boost, displaying the maximum peaks after one week, at 28 dpi (*p* < 0.0001), showing the highest response after stimulating with Lv17/WB/Rie1. A significant drop in the responses was observed just before the challenge (35 dpi), although both responses increased again after the challenge, showing a similar trend between both viral stimuli. Increases in the number of IFNγ responding cells were significant from 42 dpi [Lv17/WB/Rie1 (*p* < 0.01) and Arm07 (*p* < 0.001)] and highly significant (*p* < 0.0001) from 49 dpi. On the contrary, no virus-specific T-cell responses were observed in the non-immunized control pigs at any time during the study.

#### 3.3.2. Analysis of Primary Cellular Sources of ASFV-Specific IFNγ and TNFα by Flow Cytometry

In parallel, we used flow cytometry to identify the cellular source of virus-specific IFNγ responses (Figure 2B–E). The flow-cytometry gating strategy defining the phenotype of the different cell populations analyzed is described in Supplementary Figure S1. CD8 T cells (Figure 2B,D and Figure 5A,D) and CD4CD8 T cells (Figure 2C,E and Figure 5B,E), defined by the CD3+CD4^neg^CD8^high^ cytotoxic phenotype and by the CD3+CD4+CD8^low^ activated/memory phenotype respectively, were identified as the main source of ASFV-specific IFNγ+ and IFNγ+TNFα+ after immunization with the Lv17/WB/Rie1 strain. In the immunized pigs, significant ASFV-specific cytokine responses (IFNγTNFα) from T cells CD8 (Figure 2B and Figure 5D) and CD4CD8 (Figure 2C and Figure 5E) were observed following ex vivo stimulation of PBMC with both viruses. Both responses were evident from 21 dpi, although they were not significant (compared with 0 dpi) until day 28. Generally, the CD4CD8 specific response observed was greater (in terms of percentages of cytokine-secreting cells) than the CD8 specific response. Similarly to what was observed with the IFNγ ELISpot, the highest cytokine responses were observed at 28 dpi, seven days after the boost, displaying both responses (CD8 and CD4CD8), with highly significant increases (*p* < 0.0001) against both viral stimuli, Lv17/WB/Rie1 and Arm07. Nevertheless, just before the challenge at 35 dpi, both responses dropped significantly, although these increased again following the challenge with Arm07. These increases, although without statistical significance, were gradual and maintained against both virus stimuli until the end of the study, being particularly evident in the case of the CD4CD8 T-cell response at 56 dpi. With regard to CD8 responses, these increased after the challenge, peaking at 49 dpi (14 dpc) against both stimuli, achieving statistical significance upon stimulation with Lv17/WB/Rie1 (*p* < 0.01) and Arm07 (*p* < 0.05). Virus-specific responses for the CD3+CD4+CD8^neg^ helper T-cell phenotype (CD4 T cells) were absent (Figure 5C,F).

#### 3.3.3. Comprehensive Functional Characterization of ASFV-Induced T-Cell Responses by Flow Cytometry in the Immunized/Challenged Animals

A more detailed flow cytometry study combining phenotypic analysis with functional assays was performed at selected time points (0, 7, 21, 28, 35, and 42 dpi) with PBMC from three representative animals in the immunized/challenge group. Following the ex vivo stimulation of PBMCs with virus, mock or medium, frequencies of CD3 T cells and the CD3+ T-cell subpopulations defined as CD3+CD4+CD8^neg^ (helper CD4), CD3+CD4^neg^CD8^high^ (cytotoxic CD8) and CD3+CD4+CD8^low^ (activated/memory CD4CD8), in combination with the simultaneous detection of intracellular IFNγ and TNFα, the cytotoxic degranulation marker CD107a and the detection of the major histocompatibility complex (MHC)-class II (SLA-class II in pigs) were studied.

(a) Changes in the frequencies of CD3 and CD3+T-cell subsets.

**Figure 3:**
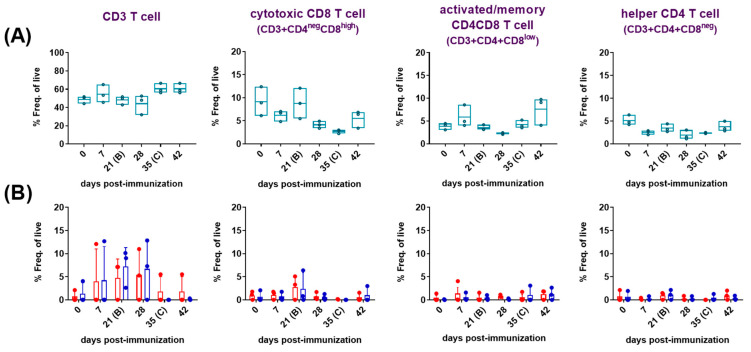
At selected days post-immunization (dpi) with Lv7/WB/Rie1 on 0 (prime) and 21 (boost) dpi and challenge with Armenia07 at 35 dpi (0, 7, 21, 28, 35 and 42 dpi), resuscitated PBMC were stimulated in vitro with medium or ASFV (Lv7/WB/Rie1 or Armenia07) or mock-virus supernatant. Phenotype and frequencies (Freq. of live) were assessed by flow cytometry. T-cell populations were defined as CD3+ (CD3 T cell), CD3+CD4^neg^CD8^high^ (cytotoxic CD8 T cell), CD3+CD4+CD8^low^ (activated/memory CD4CD8 T cell) and CD3+CD4+CD8^neg^ (helper CD4 T cell). (**A**) Data of cellular populations on live, singlet lymphocytes after medium stimulation. Spontaneous/basal responses to medium in the immunized pigs. Floating bars of individual data and a line at the mean of the three pigs are shown for each time point. (**B**) Data of cellular populations on live, singlet lymphocytes after virus stimulation and corrected against mock. Responses to Lv7/WB/Rie1 (red dots and box) and Armenia07 (blue dots and box) in the immunized pigs. Individual and mean data ± SD from three pigs are shown for each time point.

The analyses were performed with PBMC from immunized/challenged pigs after priming immune responses with medium or virus (Figure 3). Basal responses to medium in the immunized animals (Figure 3A) evidenced a moderate and transient, yet not significant, increase in frequencies of CD4CD8 cells 7 days after the prime immunization. On the contrary, CD8 and CD4 frequencies experienced a clear decrease at 7 dpi. All the CD3+ subsets returned to pre-immunization values at 21 dpi, except for the helper CD4 cells, which remained at lower levels from 7 dpi and during all the study. Although not statistically significant, additional changes were noticed after the boost (28 dpi). Thus, a second inoculation of Lv17/WB/Rie1 induced a new drop in CD8 cells and a small and transitory decrease in CD4CD8 and CD4 cells. CD3 cells rose from 35 dpi onwards, remaining high and above pre-immunization levels until the end of study. However, none of the three CD3 subsets studied exhibited a similar trend, except for CD4CD8 and, to a lesser extent CD8 at 42 dpi, which increased again after the challenge. Nevertheless, these changes were not statistically significant compared to previous values. On the other hand, the frequency of specific CD3 T cells in response to recall antigen increased most markedly between 7 and 28 dpi (Figure 3B), showing less individual variability and reaching the highest numbers, on average, between 21 and 28 dpi against both virus stimuli. At 21 dpi, a moderate, albeit not statistically significant, increase in the frequency of virus-specific CD8 T cells was observed. No noticeable changes in the virus recall response were observed among the other populations studied.

(b) Assessment of the expression of IFNγ, degranulation marker CD107a, MHC-class II and the co-expression of TNFα.

**Figure 4:**
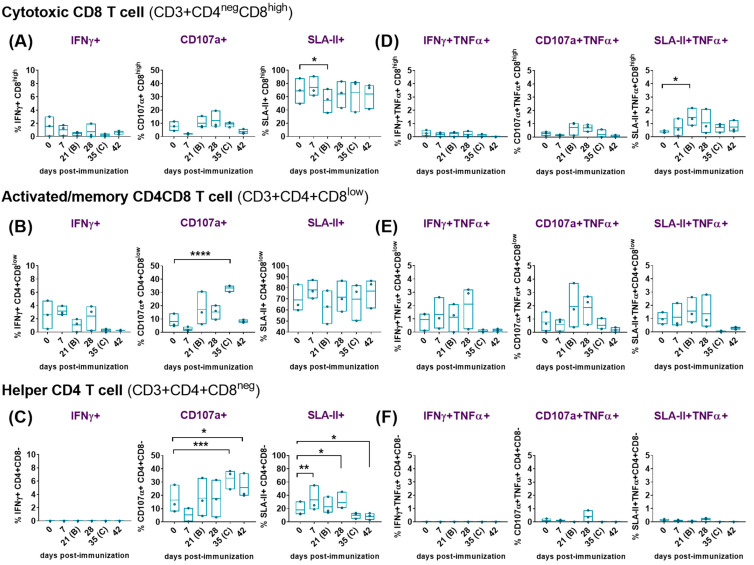
Evaluation of IFNγ response, cytotoxicity (CD107a degranulation marker) and expression of porcine class II major histocompatibility complex (SLA-II) of cytotoxic CD8, activated/memory CD4CD8 and helper CD4 T cells following immunization with Lv7/WB/Rie1 on 0 (prime) and 21 (boost) dpi and after challenge with Armenia07 (35 dpi). Responses of resuscitated PBMCs to stimulation with medium were assessed by flow cytometry at selected days post-immunization (dpi) (0, 7, 21, 28, 35 and 42 dpi). T-cell populations were defined as CD3+CD4^neg^CD8^high^ (cytotoxic CD8), CD3+CD4+CD8^low^ (memory CD4CD8) and CD3+CD4+CD8^neg^ (helper CD4). Single expression of intracytoplasmic IFNγ, CD107a and SLA-II was assessed for CD8 (**A**), CD4CD8 (**B**) and CD4 (**C**) T cells. Representative data for each cellular population on live, singlet lymphocytes after medium stimulation are presented as % IFNγ+, %CD107a+ and % SLA-II+. Double expression of intracytoplasmic IFNγ and TNFα, CD107a and TNFα, and SLA-II and TNFα was assessed for CD8 (**D**), CD4CD8 (**E**) and CD4 (**F**) T cells. Representative data for each cellular population on live, singlet lymphocytes after medium stimulation are presented as % IFNγ+TNFα+, % CD107a+TNFα+ and % SLA-II+TNFα+. Spontaneous/basal responses to medium in the immunized pigs. Floating bars of individual data and a line at the mean from three pigs are shown for each time point. Significance is indicated by the following: * (*p* < 0.05), ** (*p* < 0.01), *** (*p* < 0.001) and **** (*p* < 0.0001).

**Figure 5:**
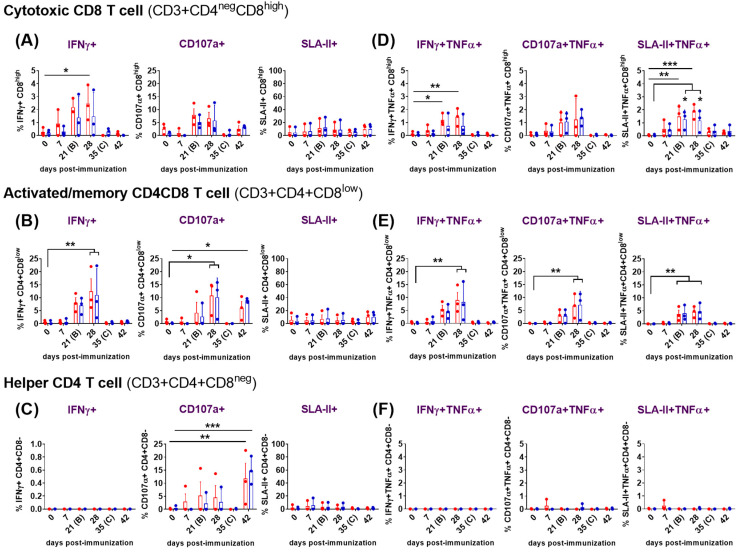
Evaluation of IFNγ response, cytotoxicity (CD107a degranulation marker) and expression of porcine class II major histocompatibility complex (SLA-II) of cytotoxic CD8, activated/memory CD4CD8 and helper CD4 T cells following immunization with Lv7/WB/Rie1 on 0 (prime) and 21 (boost) dpi and after challenge with Armenia07 (35 dpi). Responses of resuscitated PBMCs to stimulation either with Lv7/WB/Rie1 (red dots and box), Armenia07 (blue dots and box) or mock-virus supernatant were assessed by flow cytometry at selected days post-immunization (dpi) (0, 7, 21, 28, 35 and 42 dpi). T-cell populations were defined as CD3+CD4^neg^CD8^high^ (cytotoxic CD8), CD3+CD4+CD8^low^ (memory CD4CD8) and CD3+CD4+CD8^neg^ (helper CD4). Single expression of intracytoplasmic IFNγ, CD107a and SLA-II was assessed for CD8+ (**A**), CD4+CD8+ (**B**) and CD4+ (**C**) T cells. Representative data for each cellular population on live, singlet lymphocytes after virus stimulation are presented as the mock-corrected % IFNγ+, %CD107a+ and % SLA-II+. Double expression of intracytoplasmic IFNγ and TNFα, CD107a and TNFα, and SLA-II and TNFα was assessed for CD8 (**D**), CD4CD8 (**E**) and CD4 (**F**) T cells. Representative data for each cellular population on live, singlet lymphocytes after virus stimulation are presented as the mock-corrected % IFNγ+TNFα+, % CD107a+TNFα+ and % SLA-II+TNFα+. Individual and mean data ± SD from three pigs are shown for each time point. Significance is indicated by the following: * (*p* < 0.05), ** (*p* < 0.01), *** (*p* < 0.001).

Simultaneous analyses of phenotype and function were performed with PBMCs from immunized/challenged pigs after priming immune responses with medium (Figure 4) or virus (Figure 5). Spontaneous/basal responses to medium in the immunized pigs (Figure 4) evidenced some changes in cytotoxic CD8 (Figure 4A,D), activated/memory CD4CD8 (Figure 4B,E) and helper CD4 (Figure 4C,F) T cells after the prime immunization in the immunized/challenge animals. Immunization induced a moderate, yet not significant, increase in baseline levels of CD4CD8 T cells expressing IFNγ alone or in combination with TNFα, mainly after the prime (7 dpi) and the boost (28 dpi). This CD4CD8 response was reduced to almost-absent levels from 35 dpi until the end of the study (Figure 4B,E). Baseline IFNγ and IFNγTNFα CD8 responses to medium were very poor and barely suffered any changes during the study (Figure 4A,D). Meanwhile, spontaneous cytokine response from CD4 T cells was absent for all the study (Figure 4C,F).

To further elucidate this response, the ability of these cells to degranulate and secrete both cytokines was investigated. All subsets of CD3 T cells studied (CD8, CD4CD8 and CD4) showed evidences of non-specific cytotoxicity after immunization, as indicated by the increase in CD107a expression from 21 dpi onward. CD107a+CD8 showed the highest increase at 28 dpi, although this increase did not reach statistical significance at any time point during the study (Figure 4A). CD107a+CD8 and CD107a+CD4CD8 cytotoxic T-lymphocytes (CTLs) simultaneously secreting TNFα also showed a similar trend (Figure 4D,E), with the CD4CD8 CTLs displaying the highest frequencies and maximal increases, on average, at days 21 and 28 (Figure 4E). Although differences observed at these time points were not statistically significant due to variability between individuals, they were more pronounced for CD4CD8 CTLs (Figure 4E). Interestingly, basal frequencies of cells expressing CD107a were significantly high for CD4CD8 (*p* < 0.0001) (Figure 4B) and CD4 T cells (*p* < 0.001) (Figure 4C) before the challenge (35 dpi), and for CD4 T cells (*p* < 0.05) also after the challenge (42 dpi) (Figure 4C). This indicated that both T-cell subpopulations exhibited high levels of cytotoxic activity even before the challenge, which may have contributed to the defense against the viral challenge.

Analysis of basal expression of the porcine MHC-class II (SLA-II) in the immunized/challenged group following the prime immunization with Lv17/WB/Rie1 revealed some significant changes. Thus, immunization led to an evident increase in the frequency of CD8, CD4CD8 and CD4 T cells that expressed this marker. This increase was observed after priming (7 dpi) and after boosting (28 dpi) in the three CD3 T-cell subsets. In all cases, the increase in SLA-II expression was most notable after the prime, particularly evident at 7 dpi, although statistical significance was observed only for CD4 at this time point (*p* < 0.01), and after the boost at 28 dpi (*p* < 0.05) (Figure 4C). SLA-II expression levels remained elevated for CD8 (Figure 4A) and for CD4CD8 (Figure 4B) after boost (from 21 dpi onwards), although it is worth pointing out that the challenge with Arm07 triggered a further rise in the percentage of SLA-II+CD4CD8 cells (42 dpi) but not in the others. The SLA-II+CD4 cells frequencies decreased markedly from 35 dpi, falling below pre-immunization levels and remaining low until the end of the study, when levels show a statistically significant decrease (*p* < 0.05) (Figure 4C). After immunization, the double positively stained (SLA-II+TNFα+) CD8 (Figure 4D) and CD4CD8 (Figure 4E) cell subsets increased from 7 to 28 dpi, peaking between day 21 and 28; however, significant differences with pre-immunization levels could be observed only for the SLA-II+TNFα+CD8 by day 21 (*p* < 0.05) (Figure 4D). At the time of the challenge (35 dpi), frequencies of CD8 and CD4CD8, which were simultaneously expressing SLA-II and TNFα, dropped almost to pre-immunization levels, which were maintained until the end of the study (Figure 4D,E). Percentages of SLA-II+TNFα+CD4CD8 at 35 and 42 dpi were even lower than the ones observed at day 0 (Figure 4E). Finally, baseline SLA-II+TNFα+CD4 responses to medium were barely detected and did not suffer any changes during the study (Figure 4F).

The detailed functional analysis of ASFV-specific responses after priming PBMCs with both viruses revealed significant findings (Figure 5). In addition to the virus-specific IFNγ+TNFα+CD8 and IFNγ+TNFα+CD8CD4 T-cell responses observed in the immunized animals after the prime immunization (described in Section 3.3.2), a high proportion of the responding cells also expressed CD107a, indicating cytotoxic activity. The ASFV-specific CD107a+CD8 CTL (Figure 5A) and CD107a+CD8CD4 CTL (Figure 5B) responses peaked between 21 and 28 dpi, with the CD8CD4 response achieving statistical significance at 28 dpi (*p* < 0.05). On day 42, a virus-specific CD8CD4 cytotoxic response was mounted against both virus stimuli, although it only reached significant difference (*p* < 0.05) compared to day 0 after priming with Arm07 (Figure 5B). A proportion of the responding CD8 CTLs and CD8CD4 CTLs also co-expressed TNF-α (Figure 5D,E), and such responses were more pronounced from 21 to 28 dpi. However, only the CD107a+TNFα+CD8CD4 response achieved a significant difference (*p* < 0.01) at 28 dpi (Figure 5E). These findings, illustrated in Figure 5, indicated robust cytotoxic and cytokine-producing responses mounted by T cells following priming with ASFV, particularly observed in the CD8CD4 subset.

With regard to CD4 helper T cells, notable cytotoxic responses were observed at 42 dpi (7 days post-challenge), when a highly significant CD4 CTL response was mounted against both Lv17/WB/Rie1 (*p* < 0.01) and Arm07 (*p* < 0.001) (Figure 5C). However, neither CD107a+ nor CD107a+TNFα+ ASFV-specific responses were clearly observed in the CD4 T cell subset before this point, indicating minimal or absent cytotoxic activity of these cells until day 42 (Figure 5C,F).

For SLA-II, a virus-specific SLA-II+CD8 response triggered by stimulation with both viruses was observed from day 7 onwards (Figure 5A). These responses, although not statistically significant, peaked between 21 and 28 dpi. Beyond this point (28 dpi), SLA-II+CD8 response decreased, although a new increase following the challenge was detected. A similar trend was observed for the SLA-II+CD4CD8 cells (Figure 5B). On the other hand, the SLA-II+CD4 response was of lower intensity and appeared earlier, showing a small peak at day 7 (Figure 5C). In line with the induction of virus-specific IFNγ+TNFα+ and CD107a+ TNFα+ expression displayed by both CD8 and CD8CD4 T cells subsets, a highly significant virus-specific SLA-II+TNFα+ response was mounted against Lv17/WB/Rie1 and Arm07 from both subsets at 21 and 28 dpi (Figure 5D,E). In particular, the specific SLA-II+TNFα+CD8 response (Figure 5D) against Lv17/WB/Rie1 achieved significant differences at day 21 (*p* < 0.01), displaying even higher differences at day 28 (*p* < 0.001). While the differences at day 21 and 28 were also significant against Arm07, these achieved lower significance (*p* < 0.05). Likewise, the SLA-II+TNFα+CD4CD8 response (Figure 5E) against both virus stimuli was statistically significant (*p* < 0.01) on both days (21 and 28 dpi). No SLA-II+TNFα+ ASFV-specific responses were observed for the CD4 subset (Figure 5F).

## 4. Discussion

In this study, intradermal immunization of domestic pigs, using a prime/boost regime with the attenuated Lv17/WB/Rie1 strain, conferred high levels of protection against virulent challenge with a related genotype II Arm07. Both strains share a 99.94% when both full-genome sequences are compared. The most notable differences were those particularly regarding the CD2 gene. The attenuated Lv17/WB/Rie1 strain carries a mutation in the CD2 gene, resulting in its non-expression of protein. This gene mutation has been associated with decreased virulence in ASFV strains [23]. Unlike intramuscular inoculation, which may lead to adverse reactions, the intradermal approach not only conferred robust protection but also reduced adverse reactions typically associated with live-vaccine candidates in domestic pigs [23,24]. Although one of the five immunized pigs developed viremia and moderate clinical signs after the first intradermal inoculation, leading to its euthanasia prior to the boost, the remaining four animals were fully protected. These four pigs developed a robust immunity which was evident after the prime immunization, characterized by the induction of ASFV-specific antibodies and virus-specific IFNγ-T cell responses. This pattern of immune response aligns with previous findings, suggesting that the combination of antibody induction with a potent virus-specific cellular response improves protection against subsequent infections [15,16,35]. Hence, this experimental model provides an excellent opportunity to study in detail the mechanisms defining the protective immune response induced by attenuated strains against the virulent ASFV challenge in natural hosts.

The mild and transient increase in body temperature observed after prime immunization with the attenuated strain, coupled with low and transient viremia, is consistent with previous observations in both domestic pigs [23,24] and wild boar [21,22]. Unlike the findings in wild boar immunized orally with repeated doses of the Lv17/WB/Rie1 strain [22], the intradermal route did not induce notable viremia or clinical signs after the booster dose. These differences could be attributed to the different routes of administration used (intradermal vs. oral). Previous studies have proven that intradermal vaccination is apparently more effective than other routes of vaccination, such as intramuscular or subcutaneous, even if the latter are administered in repeated doses [36,37,38]. Although vaccination by these routes may be equally immunogenic, the dose may be reduced when the vaccine is administered intradermally [39]. Dermis is rich in resident dendritic cells (DCs), especially Langerhans cells and dermal DCs, and although plasmacytoid DCs (pDCs) are rare in skin, they quickly infiltrate this organ during inflammation. It is known that pDCs become stimulated by the virus to produce type I IFN during acute ASFV infections (reviewed in [40]), a cytokine that seems to be crucial in innate protection against some attenuated ASFV strains [20,41]. Therefore, as has been suggested in other immunization studies carried out against hemorrhagic viruses such as Ebola [42], intradermal immunization would trigger enhanced adaptive immune responses by recruiting more dermal DC subsets to the inoculation site, including pDCs. It would increase the chances of success against subsequent infections even by using reduced or single doses during immunizations; this is a hypothesis that requires further studies with ASFV in domestic pigs and wild boar.

An increase in circulating levels of all cytokines studied was observed after the challenge in the non-immunized control pigs. This event, also known as “cytokine storm,” is directly associated with severe disease caused by virulent strains when animals lack protection [43,44]. Unlike controls after the challenge, where IL-8 levels increased significantly in parallel with the development of clinical signs and viremia, IL-8 levels in the immunized animals remained relatively stable after the initial peak following the first immunization (between 3 and 10 dpi), with minimal fluctuations observed after the booster and challenge. Although studies on IL-8 are contradictory, and often fail to observe a significant modulation of this chemokine following infection with virulent or attenuated ASFV isolates, our findings align with previous studies indicating an increase in circulating levels of IL-8 following infection with the virulent Arm07 strain [45]. The early and controlled increase of IL-8 in immunized pigs suggests an in vivo modulation of circulating IL-8 after immunization with the attenuated Lv17/WB/Rie1 strain, which appears to be correlated with protection against ASFV.

Similarly, IL-10 levels remained minimally affected in the immunized/challenged group, except for some animals that exhibited a mild increase shortly after the first immunization (3 dpi). In contrast, in the control group, a substantial increase in IL-10 was observed, consistent with the rise in IL-8 after the challenge, indicating, in line with other studies (reviewed in [44]), a direct correlation between elevated levels of both cytokines and an exacerbated and/or uncontrolled inflammatory immune response after the Arm07 challenge.

The controlled and early increases in IL-10, cytokine with a potent and broad anti-inflammatory activity, have also been described in wild boar experimentally immunized with Lv17/WB/Rie1 [46], as well as in domestic pigs experimentally inoculated with some attenuated ASFV vaccine strains [18,20,47]. Thus, our results are consistent with the premise that a controlled and early increase in IL-10 may contribute to controlling viral replication and dampening the exacerbated inflammatory response that often leads to fatal outcomes during ASFV infections. The simultaneous decrease in circulating levels of TNFα and IFNα, two cytokines with important pro-inflammatory functions, which are normally associated with tissue damage and the appearance of clinical signs [44,48], showed evidence of a restrained inflammatory response in the immunized animals. In summary, the transient increase in IL-8 and IL-10 observed in some pigs immunized with Lv17/WB/Rie1 might suggest a role in survival, by contributing to controlling the spread of the attenuated virus and the inflammatory response. Thus, controlled and transient peaks of these two cytokines in serum, but especially IL-8, could be good markers of protection as well as of the favorable evolution of ASFV infection.

IFNγ plays a pivotal role in inducing and modulating immune responses, but it remains unclear whether protection against ASFV is linked to IFNγ production. A study conducted in domestic pigs reported a significant rise in circulating IFNγ, as well as TNFα, seven days after immunization with the attenuated vaccine candidate HLJ/18-7GD [14]. Similarly, significant increases were observed at 28 dpi in wild boar orally inoculated with Lv17/WB/Rie1 [46]. However, other studies did not find remarkable changes in circulating levels of IFNγ following the immunization of pigs with different attenuated ASFV isolates [20,49], including the Lv17/WB/Rie1/d110-11L and Lv17/WB/Rie1 ASFV strains inoculated intramuscularly [24]. In our study, although intradermal immunization with Lv17/WB/Rie1 induced in some animals detectable increases of circulating IFNγ and TNFα after the boost and challenge, our results do not prove the existence of a direct association between protection and the induction of these serum cytokine peaks; however, this hypothesis certainly cannot be excluded.

Regarding the phenotypic characterization of the T-cell responses, the different subpopulations of PBMCs in the immunized animals suffered changes in their frequencies throughout the experiment. CD3 T lymphocytes, and particularly activated/memory CD4CD8 cells, experienced a moderate and transient increase 7 days after immunization with Lv17/WB/Rie1, while, at the same time, cytotoxic CD8 and helper CD4 T cells experienced a clear drop in their frequencies. Significant increases in CD4CD8 cells have been reported as early as 4 days after immunization, with the attenuated genotype I vaccine candidates OURT88/3 and Benin ΔMGF [47]. Fluctuations in cytotoxic CD8 cells after immunization with both vaccine strains have also been reported [47]. However, other authors did not observe changes in pigs immunized with OURT88/3 prior to the challenge [35]. A progressive decrease in circulating CD4 helper T cells has also been described with the same genotype I vaccine strains listed above, in both protected and unprotected pigs [35,47]. In our study, all subsets of CD3 cells increased again after the challenge, mainly CD4CD8, and moderately in the case of the CD8 cells and, to a lesser extent, the CD4 cells. Increases in the number of both CD8+ T cells (mainly double-positive), have also been reported following the virulent challenge in pigs that became protected after immunization with OURT88/3 [35]. Immunization with Lv17/WB/Rie1 also induced a virus-specific CD3 proliferative response between 21 and 28 dpi and, although none of the T-lymphocyte subpopulations analyzed showed significant changes, this increase in circulating T cells provides evidence of activation of the adaptive cellular immune response following immunization capable of responding to the challenge with Arm07.

A peculiarity of the porcine immune system is the high expression of MHC class II (SLA-II) DR in resting lymphocytes, although the use of this marker, in combination with CD8α, has been demonstrated to be of great help in identifying activation in porcine helper T cells [50]. Previously to immunizing the animals, circulating CD4 helper T cells lacked CD8 expression and had variable expression of the SLA-II, phenotype that matched the normal description of resting helper T cells. CD4CD8 and CD8 T cells, however, expressed high levels of SLA-II, which is in line with the description carried out of these T-cell subsets in the blood of healthy pigs [51]. Immunization with Lv17/WB/Rie1 led to the transient upregulation of the SLA-II surface protein in circulating CD8, CD4CD8 and CD4 T cells after the prime and boost; however, these increases were d significant only for the CD4 T cells. The transitory upregulation of SLA-II protein expression induced after immunization with Lv17/WB/Rie1 would indicate an increase in antigen presentation phenomena and would confirm the existence of regulatory mechanisms that activate an adaptive immune response. Due to the fact that co-expression of SLA-II and CD8α appears strongly associated in TCR- αβ T lymphocytes [50,51], it was not surprising that SLA-II expression remained high in both CD8-positive T cells for all the study.

The simultaneous production of different cytokines or effector molecules at the single T-cell level has been proposed to be a hallmark of protective immune responses. For this purpose, we aimed to identify potential multifunctional virus-specific T cells. The correlation between virus-specific IFNγ-producing cells and protection has been described in some in vivo studies by using different techniques such as ELISA, ELISpot assay or flow cytometry [9,16,52]. In other studies, in which the authors carried out a phenotypic characterization by flow cytometry of IFNγ-producing cells in vaccinated pigs, they did not find a clear relationship between the protection and induction of IFNγ-specific T cells. However, they could not rule out a possible protective role for these cells [15,35,47]. The combined expression of two cytokines, such as IFNγ and TNFα, is a good indicator of the quality of the responses. Our results showed a high proportion of CD8 and CD4CD8 cells which, along with IFNγ, also co-expressed TNFα, with CD4CD8 cells exhibiting the highest virus-specific IFNγ+TNFα+ response throughout the study. Although a specific CD8 T-cell response was detected in some animals as early as 7 dpi, the induction of both responses became clearer between 21 and 28 dpi, showing the highest level of significance at 28 dpi (7 days after the boost). The induction of elevated percentages of ASFV-specific polyfunctional memory T cells, i.e., IFNγ+TNFα+ CD4CD8 T cells in pigs immunized with the BA71ΔCD2 deleted mutant, a vaccine candidate that conferred protection against the virulent challenge with genotype II Georgia2007/1 strain, has also been described recently [16]. In our study, the high levels of circulating IFNγ+TNFα+ CD4CD8 cells detected 7 days after boosting (28 dpi), in the absence of a recall virus antigen, would also suggest that CD4CD8 T cells might be involved in the spontaneous increases of these cytokines detected in sera at this time point. Hence, intradermal immunization with Lv17/WB/Rie1 induced a robust ASFV-specific IFNγ T-cell response, which was clearly detectable after the first immunization at 21 dpi, prior to the booster, where CD4CD8 and CD8 T cells were identified as the main cellular sources of virus-specific IFNγ and TNFα. These results demonstrate the correlation between the induction of virus-specific CD4CD8 and CD8 T cells and protection against subsequent infections with both attenuated and virulent strains of ASFV genotype II.

One of our aims was to study the role of specific cytotoxic T-lymphocytes (CTLs) in protection against subsequent ASFV infection. Although surface expression of CD8 has traditionally been attributed to cytotoxic functions, it is important to note that not all CD8 cells exhibit this capability [53]. Therefore, the inclusion of markers indicating cytotoxicity, such as perforin or CD107a, may be useful in defining CTL subpopulations. The CD107a assay has been used to study cytotoxic degranulation associated with loss of perforin in porcine T cells following antigenic stimulation in other porcine viral diseases such as classical swine fever [34], porcine respiratory and reproductive syndrome (PRRS) [33,54] or swine influenza A [55]. Although CD4+CD8+ and CD8 T-cell cytotoxic activity, demonstrated by the detection of perforin expression, has been described during experimental ASFV infections [13,16,53,56], to our knowledge, this is the first study reporting the use of CD107a assay to identify CTLs in ASFV-infected pigs. Immunization induced a variable, although progressive, increase in T cells with cytotoxic function in the blood, as demonstrated by the detection of the CD107a marker. Mainly in the case of CD4CD8 CTLs, this increased cytotoxic activity was accompanied by a marked and spontaneous secretion of cytokines and by an increased expression of SLA-II. Only in the case of CD4 CTLs was their progressive increase in the blood not accompanied by simultaneous cytokine production, although this increase in circulating CD4 CTLs did appear to be associated, only after the boost (at 28 dpi), with an up-regulation of SLA-II expression on CD4 helper T cells. Similarly, upon virus stimulation, IFNγ+ and IFNγ+TNFα+ producing T cells were limited mainly to cytotoxic CD4CD8, and also to cytotoxic CD8. Taken together, these results confirm the important role of CD4CD8 T cells during the early stages of infection with attenuated ASFV strains in stopping viral replication, but also their key role in mounting an effective adaptive immune response that induces protection against subsequent infections. The generation of an important subpopulation of antigen-experienced CD4CD8 T cells during the induction of the adaptive immune response was also confirmed. Furthermore, a second subset of memory CD8 T cells (CD4^neg^CD8^high^) was also identified. These cells were able to proliferate quickly after antigen re-encounter, as indicated by the quality of these virus-specific multifunctional CD4CD8 and CD8 T responses elicited upon stimulation with both viruses at day 21 and especially at 28 dpi. Beyond 28 dpi, only CD4CD8 CTLs and CD4 CTLs remained very significantly elevated. However, after the challenge, while CD4CD8 CTLs declined dramatically, CD4 CTL frequencies remained markedly elevated. The strong correlation between CD4 CTL appearance and control of infection with Arm07 suggested an important protective role of this subset in the control of early replication and infection with a virulent virus in previously immunized pigs. It is noteworthy that, although the specific cytokine secretion by these T-cell subsets after the challenge was weak, all of them (mainly CD4, followed by CD4CD8) were able to mount significant virus-specific CTL responses against both viral stimuli, indicating also the correlation between CD4 and CD4CD8 CTL subsets and protection against subsequent infection with a virulent genotype II isolate. The inclusion of additional markers, such as CD25, might help to better differentiate memory subpopulations among these CTL subsets.

Recent evidence highlights the potential role of CD4 CTL in controlling and protecting against viral diseases in pigs, particularly in the context of porcine respiratory and reproductive syndrome virus (PRRSV) infections [54,57]. These studies suggest that, in addition to the high levels of PRRSV-specific CD4CD8 CTLs acquired by vaccination or previous infection, elevated levels of PRRSV-specific CD4 CTLs are crucial in host defense against subsequent infections, even in the absence of neutralizing antibodies [54,57]. While CD4 CTLs are unlikely to replace the function of CD8 CTLs or CD8CD4 CTLs, CD4 cytotoxic activity contributes to immune responses by targeting antigen-presenting cells (APCs,) via the MHC class II pathway. In mice and humans, CD4 CTL presence is associated with chronic viral infections, autoimmune diseases, and cancer, attributing to them important antiviral functions and suggesting their potential importance during adaptive cytotoxic immune responses [58]. The induction of CD4 CTL responses targeting APCs may be particularly relevant in scenarios where CD8 CTL responses are insufficient or compromised due to sustained antigenic stimulation. It is possible that the immunization regimen used in our study, involving a prime and boost, led to CD8 cytotoxic-cell fatigue, potentially explaining the relatively weak specific responses observed just before the challenge. Additionally, viruses can evade the host immune system by down-regulating MHC class I expression in infected cells, hindering T-cell recognition of viral antigens. Although this evasion mechanism has not been confirmed during in vivo infections with virulent ASFV isolates, it has been demonstrated in vitro, implicating the viral protein EP153R [59]. Thus, the increased frequency of CD4 CTLs observed after the challenge in our immunized/challenged pigs may play a crucial role in combating de novo infection with virulent ASFV, potentially through CD4 CTL-mediated killing of infected APCs, such as monocyte/macrophages, which are primary target cells for ASFV. Further elucidation of the mechanisms underlying CD4 CTL differentiation could inform the development of more effective ASFV vaccines.

## 5. Conclusions

Intradermal immunization of domestic pigs using a prime/boost regime with the Lv17/WB/Rie1 strain conferred high levels of protection against the virulent challenge with Arm07. The transient increase in IL-8 and IL-10 in serum observed after immunization might be correlated with survival. Protection was also associated with a robust ASFV-specific polyfunctional memory T-cell response, where CD4CD8 and CD8 T cells were identified as the main cellular sources of virus-specific IFNγ and TNFα. In parallel to cytokine response, these T-cell subsets also showed specific cytotoxic activity as evidenced by the increased expression of the CD107a degranulation marker. Along with virus-specific multifunctional CD4CD8 and CD8 T-cell responses, the increased levels of antigen experienced cytotoxic CD4 T cells observed after the challenge in immunized pigs might also contribute to controlling virulent infection by killing mechanisms targeting infected antigen-presenting cells. Future studies should elucidate whether the memory T-cell responses evidenced in the present study persist and provide long-term protection against further ASFV infections.

## Figures and Tables

**Table 1:** ASFV detection in tissues determined by real-time PCR in domestic pigs intradermally immunized with the Lv17/WB/Rie1 and in the control unvaccinated group (average of pigs 11 and 15). Gray indicates the positive virus (VI) isolation results after three passages in porcine blood leukocytes (PBLs) in the absence of hemadsorption (HAD). Red indicates the positive HAD result indicating the presence of Arm07 ASFV in the immunized group.

ID Domestic Pig/Tissue	Pig 6 (D65/30)	Pig 8 (D65/30)	Pig 9 (D64/29)	Pig 10 (D64/29)	Unvaccinated (D7)
Liver	No Ct	No Ct	No Ct	No Ct	17.78
Lung	No Ct	No Ct	33.7	35.7	19.29
Kidney	36.98	No Ct	No Ct	No Ct	21.9
Heart	No Ct	No Ct	37.5	36.8	22.56
Spleen	No Ct	No Ct	No Ct	No Ct	18.09
Tonsil	No Ct	37.8	No Ct	34	19.29
Renal LN *	39.05	No Ct	No Ct	No Ct	19.19
Retropharyngeal LN	No Ct	No Ct	No Ct	No Ct	19.46
Gastro-hepatic LN	No Ct	No Ct	No Ct	34.6	20.22
Mesenteric LN	No Ct	No Ct	No Ct	39.2	19.62
Mediastinal LN	33.93	No Ct	No Ct	37.8	20.10
Inguinal LN	No Ct	No Ct	38.4	No Ct	19.64
Submandibular LN	No Ct	No Ct	No Ct	36	19.07
Splenic LN	No Ct	No Ct	No Ct	35.3	20.31
Popliteal LN	39.65	No Ct	39.6	36.3	18.73
Bone marrow	No Ct	No Ct	No Ct	No Ct	18.96
Diaphragm	39.95	No Ct	37.8	32.3	24.81
Front left IA **	35.85	No Ct	No Ct	34.2	21.81
Front right IA	No Ct	No Ct	No Ct	29.8	23.97
Back left IA	No Ct	No Ct	No Ct	36	22.75
Back right IA	No Ct	No Ct	No Ct	37.3	24.28
TOTAL PCR POS.	6/21 (28.5%)	1/21 (4.8%)	5/21 (23.8%)	14/21 (66.6%)	21/21 (100%)
TOTAL VI POS.	1/21 (4.8%)	0/21 (0%)	0/21 (0%)	5/21 (23.8%)	21/21 (100%)

* Lymph node; ** articular cartilage. No Ct = undetectable.

## Data Availability

The data presented in this study are available in this article and Supplementary Materials.

## Supplementary Materials

The following supporting information can be downloaded at: https://www.mdpi.com/article/10.3390/vaccines12040443/s1, Figure S1: Flow cytometry gating strategy used for phenotyping and to interrogate immune responses.
