# Supplementary material for: Characterization of the Protective Cellular Immune Response in Pigs Immunized Intradermally with the Live Attenuated African Swine Fever Virus (ASFV) Lv17/WB/Rie1
Source: Vaccines (Basel). 2024 Apr 20;12(4):443. doi: 10.3390/vaccines12040443 (PMC11054368; doi:10.3390/vaccines12040443)
Supplement: Supplementary file 1 [file vaccines-12-00443-s001.zip › vaccines-2927631-supplementary.pdf]

Supplementary Figure S1

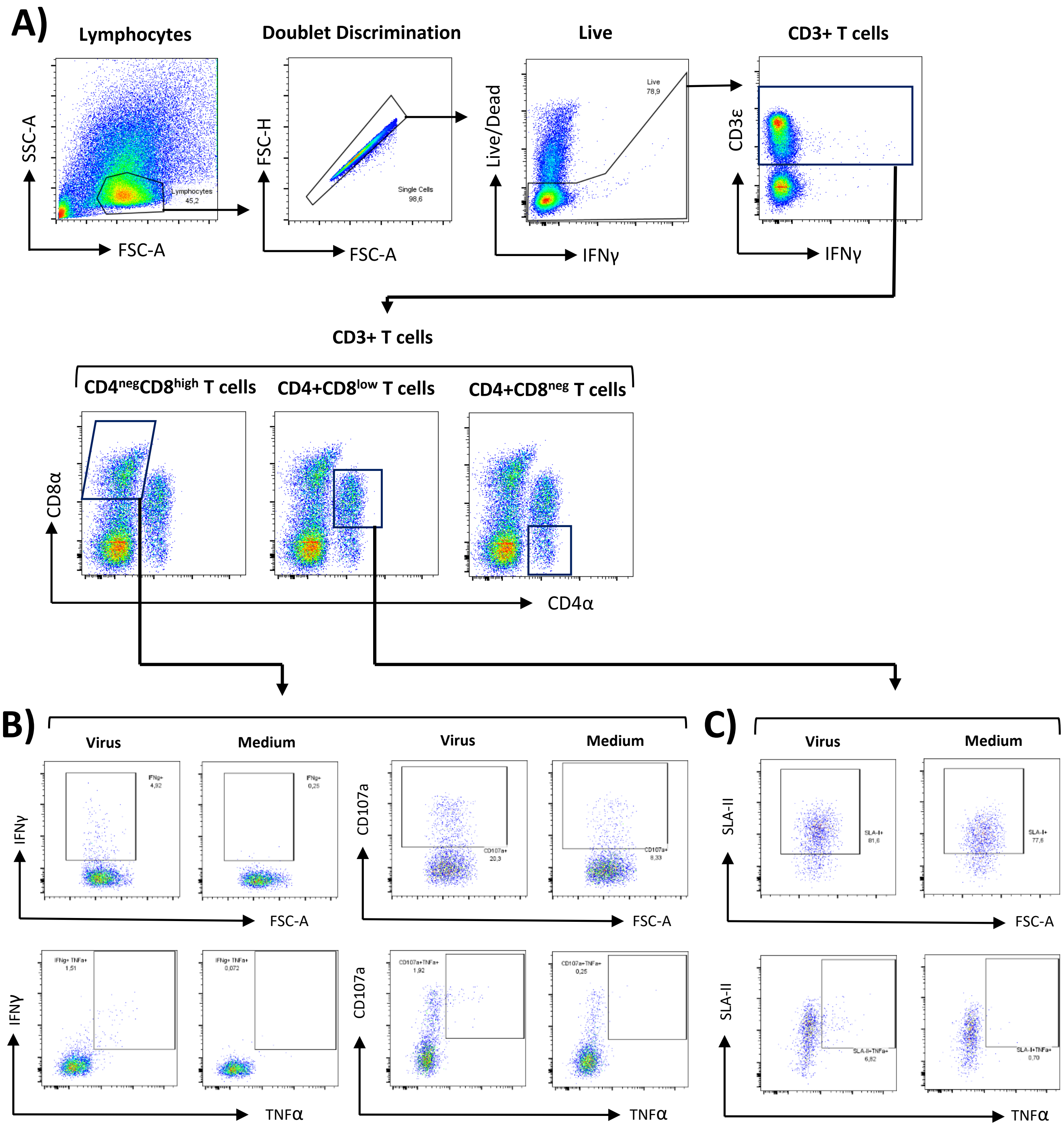

Fig S1.- Flow cytometry gating strategy for phenotyping and to interrogate immune responses. A) Representative dot plots with initial gates for lymphocytes, double discrimination and live events with subsequent gates to discriminate CD3 T cells and CD3+ T subsets. CD4^{neg}CD8^{high}, CD4+CD8^{low} and CD4+CD8^{neg} T cells discrimination based on CD3ε, CD4α and CD8α markers. B) Representative data of single expression of IFNγ+, double expression of IFNγ+TNFα+, single expression of CD107a+ and double expression of CD107a+TNFα+ on singlet, live CD3+CD4^{neg}CD8^{high} T cells after virus stimulation versus medium. C) Representative data of single expression of MHC-II+ (SLA-II) and double expression of SLA-II+ TNFα+ on singlet, live CD3+CD4+CD8^{low} T cells after virus stimulation versus medium. Final gates define percentages of single or double expression of the markers studied for each of the cell populations analyzed.
